# Changes of Mineralogical Properties and Biological Activities of Gypsum and Its Calcined Products with Different Phase Structures

**DOI:** 10.1155/2021/6676797

**Published:** 2021-03-09

**Authors:** Kaiyang Liu, Shu Han, Wei Gao, Ya'nan Tang, Xitao Han, Ziqin Liu, Liyuan Bao, Meiru Zhi, Hongyue Wang, Yingli Wang, Hong Du

**Affiliations:** ^1^School of Chinese Materia Medica, Beijing University of Chinese Medicine, Beijing 102488, China; ^2^School of Traditional Chinese Medicine, Beijing University of Chinese Medicine, Beijing 102488, China; ^3^Shanxi University of Chinese Medicine, Jinzhong, Shanxi 030619, China

## Abstract

Raw gypsum (RG) and calcined gypsum (CG) are widely used in traditional Chinese medicine (TCM). RG is usually taken orally to resolve heat and diminish inflammation, while CG is only used externally to treat ulcerations and empyrosis. Calcination at different temperatures, three phase CG structures, namely, bassanite, anhydrite III, and anhydrite II, may be generated. We herein investigated the relationship between the phase structure and the efficacy of CG and the optimum phase structure for CG. RG has a compact structure, small pore size, weak anti-inflammatory effect, but no antibacterial effect, and has almost no effect on the repair of scalds. CG150 (bassanite) has a loose texture, large pore size and specific surface area, and certain antibacterial and anti-inflammatory effects, but it has a poor repair effect on scalds. CG750 (anhydrite II) has a compact structure, small pore size and specific surface area, and low antibacterial and anti-inflammatory effects, but it has a certain repair effect on scalds. Only CG350 (anhydrite III) has good performance in texture, pore size, specific surface area, antibacterial, anti-inflammatory, and scald repair. Our research has proved that the mineral properties and biological activities of CG are different due to different phase structures. CG350, namely, anhydrite III, is considered by our research to be the optimal phase structure as CG.

## 1. Introduction

Gypsum is a monoclinal crystal mineral. Its main component is CaSO_4_▪2H_2_O [1]. Referred to as “Shi Gao” in Chinese, it has been used as a mineral medicine in China for thousands of years. Gypsum was first recorded in China's earliest materia medica book called “Shen Nong's Classic Materia Medica” (“Han” dynasty), and it is still widely used in the clinical practice of traditional Chinese medicine in modern times.

In TCM, gypsum can be used in two forms: raw gypsum (RG) and calcined gypsum (CG) [2], which are very different in their functions and usage. RG is usually used in decoction for oral administration, treating high fever, headache, and various inflammations [3, 4]. However, CG is the only medicine for external application, treating empyema, ulcers, and empyrosis [2]. According to Chinese Pharmacopeia requirements, CG should be calcined at a high temperature until it is crispy and easily crushed. However, the optimal calcination temperature for preparing CG is not specified. Studies have shown that three different phase structures, namely, bassanite, anhydrite III, or anhydrite II [5–7], will be produced when calcined at different temperatures. Previous studies only focused on changes of Ca^2+^ or whether the crystal water was utterly removed before and after calcination [8]. As far as we know, the changes of mineral properties of CGs with different phase structures or their antibacterial, anti-inflammatory, and scald treatment effects have never been studied hitherto.

CG is usually used to treat scald, with exudate absorption, wound healing, and muscle regeneration. According to TCM theory, CG needs to be calcined to opaque, crisp, and reddish. In the process of producing CG, colour and texture are essential appearance indexes to control product quality. Microstructure and pore size are often considered as mineralogical properties related to the exudate absorption ability of CG. When the scald occurs, wounds often suffer from microbial infections in untreated situations. After wounds infection, toxic molecules and metabolites produced by persistent inflammatory and bacterial and immune responses adversely affect wound repair [9]. According to related studies, *E. coli*, as a Gram-negative bacterium, is a relatively common strain of scald infection, which secretes toxins (exotoxins) that affect tissue repair [10]. During the inflammatory reaction stage of scald, there are often many overexpressed inflammatory factors, such as interleukin-1*β* (IL-1*β*) and nitric oxide (NO), which inhibit wound healing [11]. So, inhibiting microbial infection and alleviating inflammation can promote wound healing. Therefore, in this study, the efficacy of CG can be comprehensively evaluated by in vitro bacteriostatic and anti-inflammatory experiments and the expression of growth factor (transforming growth factor, TGF) related to animal scald.

To clarify which phase structure is most suitable for being included in the clinical practice, we investigated the mineralogical properties and biological activities of CGs calcined at different temperatures (150°C, 350°C, and 750°C) and RG. Fourier transform infrared spectroscopy (FTIR) and X-ray diffraction (XRD) were used to confirm the phase structure and phase composition. Scanning electron microscopy (SEM) was applied to detect the variation of microstructure. A surface area porosity analyzer (SAPA) was used to measure the pore volume and surface area, and a colorimeter was conducted to qualify the colour. In vitro, the bacterial experiment was carried out to detect the bacteriostatic activity, and a cell experiment was implemented to detect the anti-inflammatory activity. In vivo, a scald animal model was constructed to detect the repairing effect of CGs with different phase structures on scaled skin.

## 2. Materials and Methods

### 2.1. Preparation of Samples

Gypsum (license number: 1903137) was purchased from Beijing Shengshilong Pharmaceutical Co., Ltd. and identified by Professor Jingjuan Wang, an expert at Beijing University of Chinese Medicine.

Preparation of RG samples: crushing GF by ultrafine pulverizer and then sieved through a 200-mesh to obtain fine powder (particle size less than 0.074 mm).

Preparation of CG samples: 10 g of RG was put into a muffle furnace (KSW-6-12 ASP, Beijing Kewei Yongxing Instrument Co., Ltd.) and calcined at 150°C, 350°C, and 750°C for 1.5 hours, respectively, to obtain CG samples named CG150, CG350, and CG750, respectively.

### 2.2. Determination of Phase Structure

#### 2.2.1. FTIR Analysis

To reveal changes in the molecular structure of RG, CG150, CG350, and CG750, RG, CG150, CG350, and CG750 were mixed with KBr at the ratio of 1 : 100 and pressed into transparent sheets, respectively. FTIR spectroscopic characterization was determined using a FTIR spectrometer equipped with a DTGS detector (MB104, ABB Bomen Co., Quebec, Canada) in the range of 4000–400 cm^−1^. Each spectrum was recorded at the resolution of 4 cm^−1^ with 64 coadded scans. Spectrum Version 5.0 software (PerkinElmer Company) was used to collect all sample data. The data were processed by OMNIC 6.0 software (Thermo Electron Corporation, Madison, WI, USA).

#### 2.2.2. XRD Analysis

To analyze the phase composition of RG, CG150, CG350, and CG750, we detected all samples by using XRD. XRD patterns were collected on a diffractometer (Rigaku D/max 2500) with a detector voltage of 30 kV and 30 mA using a CuKa radiation source, and the scan speed was 8°min^−1^ with the 2*θ* range of 10–80°.

### 2.3. Mineralogical Properties Analysis

#### 2.3.1. SEM Observation

The samples' microstructure was observed utilizing field emission scanning electron microscopy (FESEM) (JSM-7001F, Japan). The powders of RG, CG150, CG350, and CG750 were evenly dispersed on the sample table with double-sided adhesive tape and then were sputter-coated with gold (Au) and observed using FESEM. Measurement conditions: scanning electron microscope resolution, 2 nm (30 KV)/3.0 nm (1 KV); acceleration voltage, 20 KV.

#### 2.3.2. Textural Properties

The nitrogen isotherms at the liquid nitrogen temperature were measured on SAPA (3H-2000, Beishide, China). The Brunauer–Emmett–Teller (BET) model [12] was used to obtain the surface area. Pore size distribution curves and average pore sizes were calculated by analyzing the adsorption branches of isotherms, based on the Barrett–Joyner–Halenda (BJH) algorithm [13].

#### 2.3.3. Colour Quantification

The colors of RG, CG150, CG350, and CG750 sample powders were measured by a colorimeter (Konica Minolta Japan, CM-5). The colorimeter consists of a measuring head, white calibration board, black calibration board, and colour management software (SpectraMagic NX). Six random measurements were taken on the samples and values of 3 reflectance coordinates: *L*^∗^ (lightness), *a*^∗^ (redness), and *b*^∗^ (yellowness) [14] were determined. The average values of 6 consecutive measurements were calculated separately.

### 2.4. Anti-Inflammatory Activity

#### 2.4.1. Preparation of Samples

Samples of RG, CG150, CG350, and CG750, 20 g each, were soaked in 100 mL of distilled water for 30 min, stirring with a glass rod, decocted for 30 min before filtration. The first portion of water extract was collected. The residue material then underwent the second and third extractions with boiling water, each for 30 minutes, and the second and third portions of the extract were collected. The three portions were combined and concentrated through rotary evaporation at 45°C. Finally, the extract was concentrated to 20 ml. The sample extract with a concentration of 1 g/ml was prepared.

#### 2.4.2. RAW264.7 Cells Culture

The RAW264.7 cells were purchased from the National Infrastructure of Cell Line Resource. The cells were cultured in Dulbecco's Modified Eagle's Medium (DMEM) medium containing 10% fetal bovine serum, 1% penicillin and streptomycin, and grown in incubators at 37°C and 5% CO_2_. DMEM was replaced every two days, and cells were allowed to subculture when they reached 80%–90% confluency.

#### 2.4.3. RAW264.7 Cells Viability Assay

Raw 264.7 cells were treated with extract solution of different concentrations of RG, CG150, CG350, and CG750 (100, 250, 500, 750, 1000, 1500, 2000, or 2500 mg/ml). After 24 h incubation, the medium was removed following incubation, and 20 *μ*L of MTT (5 mg/mL) was added into the wells and incubated for another 4 h. Finally, 150 *μ*L of DMSO was added, and the absorbance at 490 nm was determined. The experiments were repeated three times independently. Cell viability = (experimental pore/blank pore) × 100%.

#### 2.4.4. NO Determination

RAW264.7 cells were seeded on 96-well plates at a density of 1 × 10^5^ cells/ml. The cells were incubated at 37°C and 5% CO_2_. RAW264.7 cells were treated with LPS (1 *μ*g/mL), LPS + quercetin (5 *μ*g/mL), LPS + RG (100, 250, or 500 mg/ml), LPS + CG150 (100, 250, or 500 mg/ml), LPS + CG350 (100, 250, or 500 mg/ml), and LPS + 750 (100, 250, or 500 mg/ml). All the agents were added at the same time, and the groups were treated for 24 h. The supernatant of cells was mixed with an equal volume of Griess reagent, and the absorbance of the mixture was measured at 540 nm. The experiments were repeated three times independently.

### 2.5. Bacteriostatic Activity

#### 2.5.1. *E. coli* Viability Assay

*E. coli* (ATCC 8739) was placed in a triangular flask containing 100 ml Luria–Bertani broth medium and cultured for 20 h in a constant temperature rotary shaker at 37°C. All samples were sterilized in an autoclave (121°C, 15 min) before microbial testing to remove any environmental bacteria. After sterilization, cool to room temperature. 1 ml of bacterial suspension (∼10^8^ CFU/ml) was added to media containing 20 g sterilized samples and cultured for 20 h at a rate of 150 r/min at a constant temperature in a rotary shaker at 37°C. The mixture of bacteria and samples cultured in Luria–Bertani medium was continuously diluted to 10^8^ times, and 100 *μ*l was taken and evenly coated on Luria–Bertani agar plate and incubated in an incubator for 20 hours. Finally, the number of colonies on the agar plate was observed.

#### 2.5.2. Morphology Observation of *E. coli*

On treatment with RG, CG150, CG350, and CG750, specific morphological changes of *Escherichia coli* (ATCC 8739) were observed by FESEM (JSM-7001F, Japan). *E. coli* was cultured in 250 ml triangular flasks with 5 g/ml RG, CG150, CG350, and CG750 for 7 and 14, respectively. At 7 and 14, respectively, 1 ml of bacterial liquid was sucked, centrifuged, and separated (8000 rpm, 10 min) to obtain the bacterial bodies, washed three times with PBS, and finally fixed with 2.5% glutaraldehyde fixative for 4 h. Then, the samples were then dehydrated by increasing ethanol concentrations (30, 50, 70, 80, 90, 95, and 100%) for 10 min each time. *E. coli* grown in the blank medium was employed as a control. The bacterial morphology was fixed with 2.5% gluta fixation fluid. After being dried in air at room temperature and coated by gold sputter, samples were examined with FESEM.

### 2.6. Scald Healing Assay

#### 2.6.1. Animals

126 SPF male Kun Ming (KM) mice weighing 20–25 g were purchased from Beijing Vital River Laboratory Animal Technology Co., Ltd. (License number: SCXK (Jing) 2016002, Beijing, China). The mice were raised in the animal feeding room of BUCM under specified conditions, i.e., temperature: 22 ± 2°C; relative humidity: 50 ± 5%; and 12/12 h light-dark cycle. All the methods and procedures of animal experiments were audited and approved by the Animal Experimental Welfare Ethics Committee of BUCM (BUCM-4-2019051002-2100).

#### 2.6.2. Treatment and Assessment of Scald Healing

The scald model was created using the method described by Said et al. [15] with some modifications. For scald wound infliction, the animals were first anesthetized with pentobarbital sodium (50 mg/kg, b. w.), followed by removal of hairs from the dorsal area of mice using a hair trimmer. The model was established by heating copper sheets to induce skin scald (200°C, 3 s) with a wound diameter of 1.5 cm on the back of mice.

Preparation of ointment: RG, CG150, CG350, and CG750 were mixed with sesame oil in the ratio of 4 : 6, respectively, and RG ointment, CG150 ointment, CG350 ointment, and CG750 ointment were prepared.

A total of 126 animals were used and divided into 7 different groups (normal group, model group, RG group, CG150 group, CG350 group, CG750 group, and positive control group, *n* = 18, respectively), each having 6 animals. The model group was treated with sesame oil and considered as vehicle control. The RG group was topically treated with RG ointment; the CG150 group was topically treated with CG150 ointment; the CG350 group was topically treated with CG350 ointment; and the CG750 group was topically treated with CG750 ointment. The positive control group was topically treated with Jingwanhong Scald Ointment (JSO) purchased from Tianjin Darentang Jingwanhong Pharmaceutical Co., Ltd. (batch number: Z12020440, Tianjin, China). The treatment was given two times a day for 21 days.

On 5, 10, and 15 days after injury, 6 animals in each group were anesthetized and euthanized by intraperitoneal pentobarbital sodium injection (150 mg/kg, b. w.). Then, the skin tissues from the wound site were excised for histological observation and immunohistochemical study. For histological and immunohistochemical analyses, tissues were fixed in 10% formalin at room temperature, and unbound fixative was removed by washing in PBS.

#### 2.6.3. Histopathological Observation

The formalin-fixed tissue specimens were dehydrated by passing through gradient alcohol of 90%, 95%, and then absolute alcohol for 5, 5, and 5 min, respectively. This was followed by removing alcohol in the xylene solution and, finally, embedded in hot paraffin to prepare blocks. Blocks of a tissue section of 5 *μ* thickness were cut with a microtome. Hematoxylin and eosin (H&E) staining was used to show the morphology of skin tissue. Slice images were captured and digitized by a microscope (Nikon Eclipse Ti-SR, Nikon, Japan).

#### 2.6.4. Immunohistochemical Analysis

On 5, 10, and 15 days after injury, the expression of IL-1*β* and TGF was detected by immunohistochemistry in wound tissue sections. Immunohistochemical reactions were performed and incubated overnight at 4°C. Then, they were incubated with a secondary antibody (DAKO, China) for 50 minutes. After adequate diaminobenzidine staining and hematoxylin counter-staining, the positive expression can be observed and recorded under the microscope. The integrated optical density (IOD) was quantitatively analyzed using ImagePro Plus software (Media Cybernetics, USA).

### 2.7. Statistical Analysis

SPSS 16.0 software was used for statistical analysis, and one-way ANOVA and Student's *t*-test were used. All data were expressed as the mean ± standard deviation. *P* < 0.05 was considered significant, and *P* < 0.01 was considered extremely significant.

## 3. Results

### 3.1. FTIR Spectroscopy Analysis

**Figure 1:**
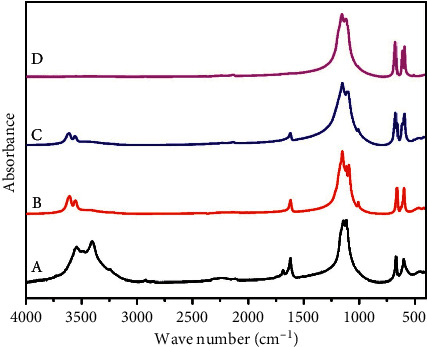
The infrared spectrum of RG (a), CG150 (b), CG350 234 (c), and CG750 (d).

As shown in Figure 1(A), the main phase composition of RG is gypsum. It is conclusive that the IR absorption bands of RG are located at around 1620 cm^−1^ and 1687 cm^−1^ (bending vibration H_2_O), 3405 cm^−1^ and 3545 cm^−1^ (stretching vibration H_2_O), 601 cm^−1^ and 668 cm^−1^ (bending vibration SO_4_^2−^), and 1115 cm^−1^ and 1141 cm^−1^ (stretching vibration SO_4_^2−^) [1, 16].

As revealed in Figure 1(B), compared with RG, the intensity of the IR absorption peak of CG150 for bending vibration (1620 cm^−1^, 1687 cm^−1^) and stretching vibration (3405 cm^−1^ and 3545 cm^−1^) of H_2_O decreased, corresponding to bassanite [17]. For CG350 (Figure 1(C)), there was a great deal of loss in absorbance at bending vibration and stretching vibration of H_2_O. CG350 FTIR peaks at 673 cm^−1^ and 1155 cm^−1^ were attributed to anhydrite III [7]. For CG750, as shown in Figure 1(D), any bending vibration and stretching vibration of H_2_O were not detected, and the IR absorption peak of CG750 was located at 594 cm^−1^, 613 cm^−1^, and 676 cm^−1^, demonstrating that the main phase of CG750 was anhydrite II [7].

### 3.2. XRD Spectroscopy Analysis

**Figure 2:**
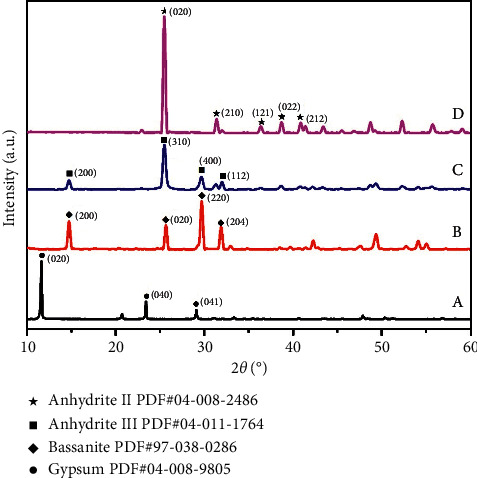
The XRD patterns of RG (A), CG150 (B), CG350 (C), and CG750 (D).

XRD was employed to analyze the phase composition changes of samples. XRD data were further processed by Jade 9 software for phase analysis. In Figure 2, the diffraction pattern of RG is consistent with the PDF file no.04-008-9805 (gypsum), showing that the main crystal structure of RG is gypsum; three prominent diffraction peaks correspond to (020), (040), and (041) crystal faces of the standard peak. The diffraction pattern of CG150 is consistent with the PDF file no. 97-038-0286 (bassanite), and four weak peaks correspond to (200), (020), (220), and (204) crystal faces, indicating that the main crystal structure of CG150 is bassanite. CG350 pattern matches the PDF file no. 04-011-1764 (anhydrite III); four weak peaks correspond to (110), (310), (400), and (112) crystal faces, illustrating that the main crystal structure of CG350 is anhydrite III. The diffraction pattern of CG750 is in keeping with the PDF file no. 04-008-2486 (anhydrite II); four weak peaks corresponding to (020), (210), (202), (220), and (212) crystal faces, illustrating that the main crystal structure of CG750 is anhydrite II. The results are in good agreement with those of FTIR.

Integrating FTIR and XRD detection results, we confirmed that the phase structures of RG, CG150, CG350, and CG750 are gypsum, bassanite, anhydrite III, and anhydrite II in order. In the next study, we will explore these four different phase structures' mineralogical properties, their antibacterial and anti-inflammatory effects, and their healing effects on scald injuries.

### 3.3. SEM Observation

**Figure 3:**
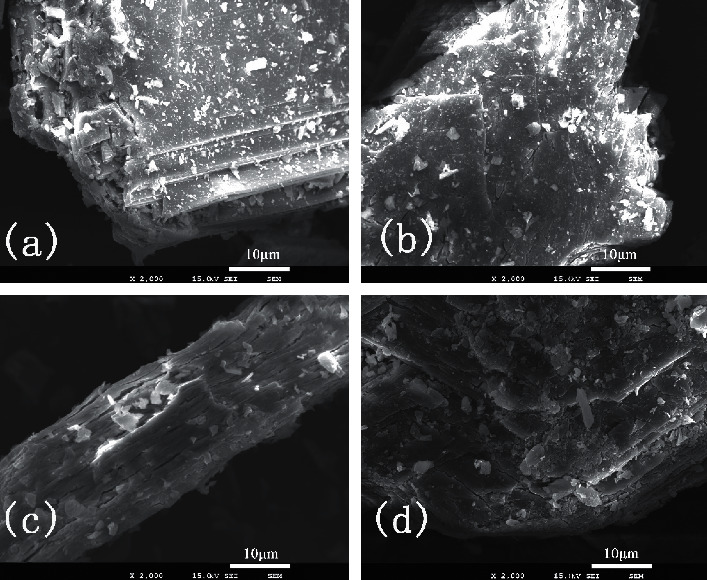
The SEM images of (a) RG, (b) CG150, (c) CG350, and (d) CG750.

SEM images clearly showed differences in texture among RG, CG150, CG350, and CG750 (Figure 3). As shown in Figure 3(a), no defects were observed on RG particles' surface, and the surface is plate-like and overlapped. While, small cracks appeared on the outside of CG150, as shown in Figure 3(b). For CG350, there are many pore structures with faults, and the marks are arranged longitudinally parallel. However, interestingly, the surface of CG750 began to appear as small and dense cracks instead of becoming looser.

According to the description of CG [2], loose and fragile means better quality. From our results, we can see that the order of the cracks on CGs from large to small is CG350, CG150, CG750, and RG; the corresponding phase structures are anhydrite III, bassanite, anhydrite II, and gypsum.

### 3.4. SAPA Measurement

**Figure 4:**
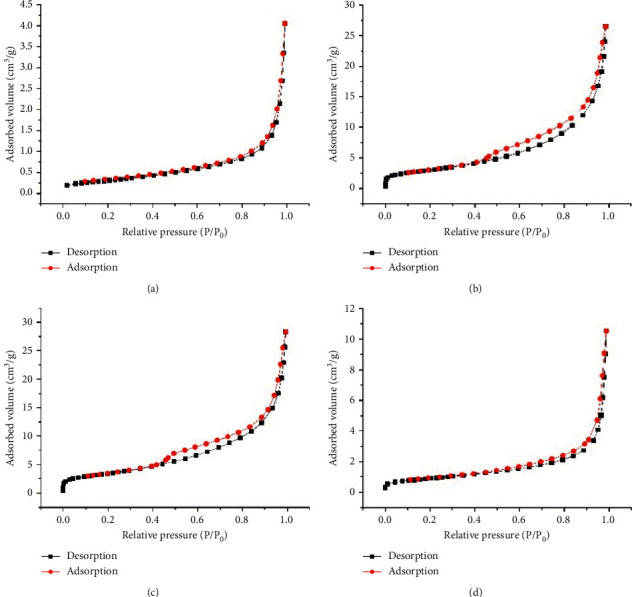
Nitrogen adsorption-desorption isotherms of (a) RG, (b) CG150, (c) CG350, and (d) CG750 powder.

The N_2_ adsorption-desorption isotherms of RG, CG150, CG350, and CG750 powders are shown in Figure 4. The N_2_ adsorption-desorption isotherms of RG and CG750 followed type III with an H1 hysteresis loop [18] in the relative pressure range of 0.8–1.0, which means that the powder has macroporous characteristics. The N_2_ adsorption-desorption isotherms of CG150 and CG350 were consistent with type IV with an H3 hysteresis loop [18] in the relative pressure range of 0.4–1.0, which means that the characteristics of the materials are mesoporous [19].

It can be seen from Table 1, as the calcination temperature of RG rises from 150°C to 750°C, the specific surface area and pore volume of the calcined products first increase and then decrease. Among RG, CG150, CG350, and CG750, CG150 and CG350 had the larger specific surface area and pore volume. The increase of the specific surface area and pore volume of CG150 and CG350 may be due to dehydration and dehydroxylation reactions in gypsum with the increase of calcination temperature [20]. When the calcination temperature is set at 750°C, due to the influence of high temperature, the internal chemical bonds of CG750 are broken, and the molecular structure is rearranged, resulting in smaller pore volume and specific surface area [21]. The relatively large surface area and pore volume mean more space to absorb exudate and keep the wound surface dry for external use. The experimental results are consistent with those of SEM observation.

### 3.5. Colour Measurement

**Figure 5:**
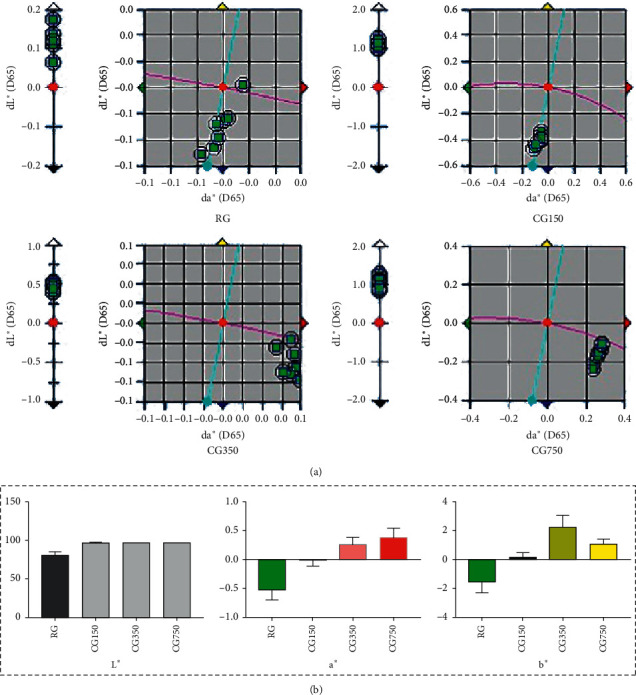
(a) Instrumental colour dL^∗^ values, da^∗^ values, and db^∗^ values' scatter diagram of RG, CG150, CG350, and CG750. (b) Instrumental colour *L*^∗^ values, *a*^∗^ values, and *b*^∗^ values of RG, CG150, CG350, and CG750 (values within each column with different letters are significantly different (*P* < 0.05)).

The colorimetric characteristics, lightness (L^∗^), redness (a^∗^), and yellowness (b^∗^) values are presented in Figure 5(b). The *L*^∗^ value results showed that the colour of all the calcined samples is lighter than the gypsum. From the detected *a*^∗^ value results, it can be found that the colour of gypsum gradually turns to red after calcination: the colour of CG750 is redder than that of CG350 and CG150, and the colour of CG350 is redder than that of CG150. CG750, CG350, and CG150's *b*^∗^ value measured with the colorimeter was significantly higher than that of RG, meaning that the colour of calcined samples was yellow, and the order of yellowness was CG350 > CG750 > CG150.

Shape, colour, smell, and texture are the traditional indicators commonly used to evaluate the quality of Chinese medicine. However, the application of them is limited due to the difficulty of quantification and the high dependence on experience. Many evaluation methods based on colour measurement have been developed in modern times to evaluate the quality of traditional Chinese medicine [22]. According to the Chinese Pharmacopoeia, CG should be white and opaque with red lustre [2]. However, it is difficult to distinguish the difference between samples with the naked eye. Colour as an appearance indicator of CG has not been determined by a quantitative method. Our colour measurement results also provide a reference for the quality control of CG.

### 3.6. Cell Experiment Results

#### 3.6.1. Effect on the Viability of RAW 264.7 Cells

**Figure 6:**
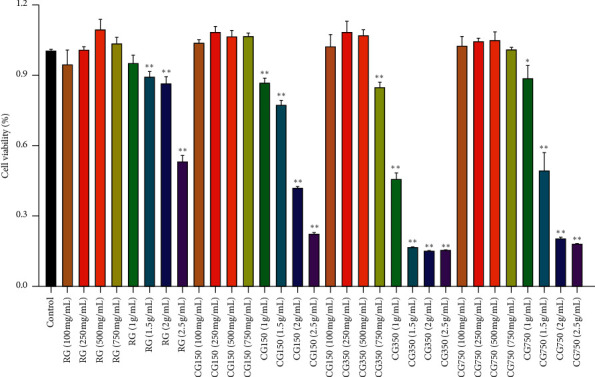
RAW264.7 cell viability. Cells were treated with different concentrations of RG, CG150, CG350, and CG750 for 24 h, and cell viability was measured using an MTT assay (^∗∗^P<0.01 and ^∗^P<0.05 vs. control).

The effects of RG, CG150, CG350, and CG750 on RAW264.7 cell viability are shown in Figure 6. On treatment with 100, 250, or 500 mg/ml, the cell viability was above 90%, implying that RG, CG150, CG350, and CG750 did not produce harmful toxicity. The LD50 of the RG group was 2853 mg/ml, that of the CG150 group was 1933 mg/ml, that of the CG350 group was 1304 mg/ml, and that of the CG750 group was 1685 mg/ml. Samples significantly decreased the cell viability at 1000 mg/ml and 2000 mg/ml (*P* < 0.01). Therefore, concentrations of 100, 250, and 500 mg/ml of samples were selected for further investigation.

#### 3.6.2. Effects on NO Production in LPS-Induced RAW264.7 Cells

**Figure 7:**
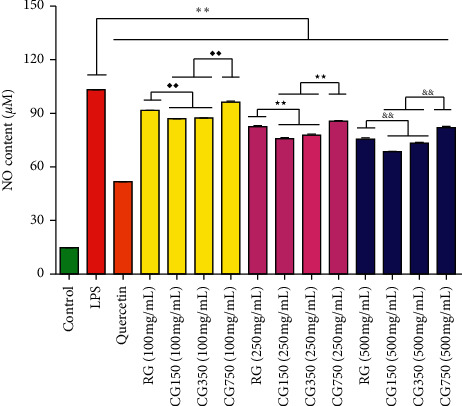
NO content of RG, CG150, CG350, and CG750. NO assay using the cells treated with LPS (1 *μ*g/ml) in the absence or presence of RG, CG150, CG350, and CG750 at different concentrations (100, 250, and 500 mg/ml, respectively) for 24 h ( ^*∗∗*^*P* < 0.01 vs. LPS; ^♦♦^*P* < 0.01 vs. CG150 (100 mg/mL) and CG350 (100 mg/mL); ^★★^*P* < 0.01 vs. CG150 (250 mg/mL) and CG350 (250 mg/mL); ^&&^*P* < 0.01 vs. CG150 (500 mg/mL) and CG350 (500 mg/mL)).

To evaluate the effect of RG, CG150, CG350, and CG750 on inflammation, the LPS-induced inflammation model was successfully established. As shown in Figure 7, compared with the LPS model group, the NO expression level in the quercetin-positive group was significantly decreased (*P* < 0.01). All samples had inhibitory effects on NO production in LPS-induced RAW264.7 cells (*P* < 0.01). The inhibitory effects of CG150 and CG350 on the release of NO were stronger than those of RG and CG750 (*P* < 0.01), and there was no significant difference between the effects of CG150 and CG350 (*P* > 0.05).

NO can be stimulated by a variety of harmful stimuli, such as pathogens, damaged cells, or irritants [23]. Excessive production of NO can induce chronic inflammation in macrophages [24, 25]. Our results showed that RG, CG150, CG350, and CG750 could dose-dependently inhibit the production of NO produced by LPS-stimulated cells, while CG350 and CG150 have a stronger effect. All these indicate that CG150 and CG350 will perform better in the inflammatory response induced by injuries such as burns or ulcers, compared with RG and CG750.

### 3.7. Bacteriostatic Experiment Results

#### 3.7.1. Antibacterial Activity

**Figure 8:**
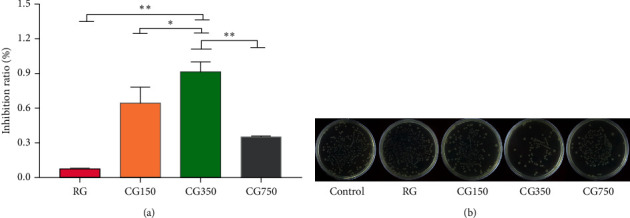
(a) Inhibition ratio of RG, CG150, CG350, and CG750 measured by a plate counting method. (b) Number of live bacteria of *E. coli* treated with culture medium, with 5 g/mL of RG, CG150, CG350, and CG750 (^∗∗^*P* < 0.01 and ^∗^*P* < 0.05 vs. CG350).

In Figure 8, there was no significant difference in the number of colonies in the RG group compared with the control group, which implied that RG has no inhibitory effect on *E. coli*. It is worth noting that the inhibition rate of CG350 on *E. coli* was 91.26 ± 7.33%. However, CG750 exhibited lower antibacterial activity, with its inhibition rate at 35.54 ± 0.57%, and the inhibition rate of CG150 was 65.50 ± 12.43%. So, CG350 performs obvious bacteriostatic advantages on *E. coli*, compared to RG, CG150, and CG750 (*P* < 0.05). The differences in the bacteriostatic effect between samples were unexpected. Our results indicated that all CG samples exhibit bacteriostatic effects after calcination, while CG samples with different phase structures showed a different antibacterial effect.

Bacterial infections are very common in burns and other traumas [26]. European Wound Management Association (2005) described that *E. coli* is an important organism causing wound infections [27]. Our results showed that CG350 has an excellent effect in inhibiting *E. coli*, which provides a piece of evidence for CG350 as a better phase structure for external use. However, the antibacterial effects of CG with different phase structures on other bacteria still need to be further studied to support this conclusion.

#### 3.7.2. Bacterial Morphological Changes

**Figure 9:**
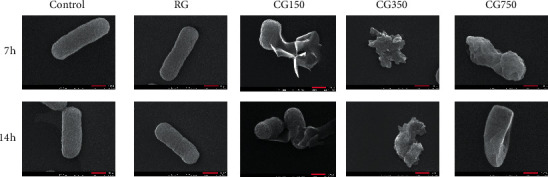
FESEM images of bacterial morphology. (a) The bacteria morphology at 7 h. (b) The bacteria morphology at 14 h.

The morphology of *E. coli* after being cultured with RG, CG150, CG350, and CG750 for 7 h and 14 h is shown in Figure 9. Under normal conditions, *E. coli* is short rod-shaped and have a smooth surface; their diameter is about 800 *n*. Compared with the control group, RG showed no effect on the cell membrane of *E. coli* at both 7 h and 14 h, while CG150, CG350, and CG750 excreted different degrees of damage to the cell membrane. Compared with normal *E. coli*, the shape of *E. coli* that interacted with CG150 and CG350 became irregular. The cell membrane was damaged and could not be kept intact. The membrane and structure of *E. coli* interacted with CG350 were more seriously damaged and broken compared with CG150 and CG750. The results of the influence on the morphology of *E. coli* are consistent with those of the antibacterial effect on *E. coli*.

Infection is one of the severe complications of trauma [28]. Over the past decades, many studies have confirmed this view that microorganisms are critical causes of delayed healing and infection of acute or chronic burn wounds [29, 30]. *E. coli* is the most common pathogen causing wound infection [31]. However, most pathogenic bacteria's resistance increases year by year with the widespread use of antibiotics [32]. It is urgent to find nontoxic materials to resist bacterial infection. CG is a widely used external medicine for wound healing in Chinese medicine. Pathogenic bacteria easily infect wounds after formation, so inhibiting the growth of bacteria is an important indicator to evaluate the efficacy of CG. In this experiment, CG showed a strong antimicrobial effect.

### 3.8. Results of Histopathological Analysis

#### 3.8.1. Histopathological Examinations

**Figure 10:**
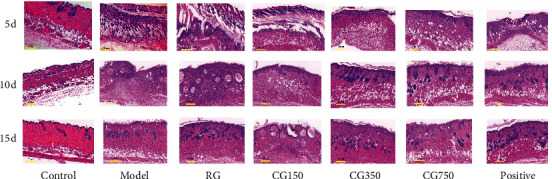
Histological appearance of scald wounds stained with hematoxylin and eosin (20x magnification, the scale is marked in the lower left corner).

As shown in Figure 10, histological observations of wounds in different groups are presented. The normal anatomy of skin tissues stained with H&E showed healthy granulation, hair follicles, epithelial cells, fibroblast cells, blood vessels, epithelial, epidermal, and dermal layers (Figure 10).

On the 5th day, in the RG group and CG150 group, skins had more severe damage to the epidermal tissue, a large number of necrotic tissue debris, pus cells, adipocyte cavities, and exudates formed by the crust structure covering, with a large number of inflammatory cell infiltration and more bleeding phenomenon. The damaged skin of the CG150 group had signs of exfoliation. In the CG350, CG750, and positive drug groups, the necrotic epidermal tissues began to recover, with tiny scabs, a small number of inflammatory cells scattered around, a small number of fibroblasts and new capillaries visible, as well as mature granulation tissue proliferation and repair, and more fibroblasts and collagen fibres visible.

On day 10, the skin of CG350, CG750, and positive drug groups recovered well and generated a large amount of mature hair follicle tissue, fat vacuoles, and arranged complete muscle tissue. The model group, RG group, and CG150 group had worse skin recovery. The model group and RG group had early hair follicle tissue generation, while the CG150 group had no hair follicle tissue generation and muscular tissue of which was also irregularly arranged.

On day 15, the skin in other groups except the CG150 group grew more mature granulation tissue, fibroblasts, collagen fibres, and many mature hair follicle tissues. Compared with other groups, the CG350 and positive drug groups had more new well-arranged hair follicle tissues and well-recovered skin blood vessels.

It is surprising in the experimental results that the effect of CG150 on wound healing is worse than that of the model group. The reason for this result may be that CG150, whose main phase structure is bassanite with strong water absorption [33], leads to excessive dryness of the wound, resulting in a secondary pus discharge from the wound [34].

#### 3.8.2. Immunohistochemical Analysis

**Figure 11:**
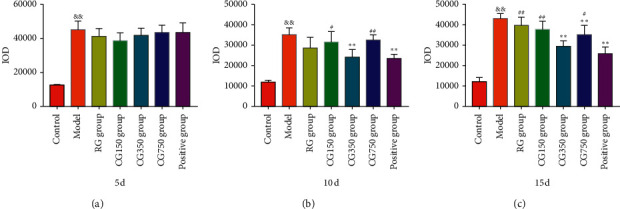
IL-1*β* expression levels in RG, CG150, CG350, and CG750 groups (^&&^*P* < 0.01 vs. the control group. ^∗∗^*P* < 0.01 vs. the model group. ^##^*P* < 0.01 and ^#^*P* < 0.05 vs. the CG350 group).

**Figure 12:**
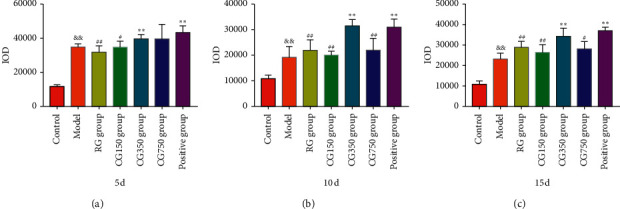
TGF expression levels in RG, CG150, CG350, and CG750 groups (^&&^*P* < 0.01 vs. the control group. ^∗∗^*P* < 0.01 and ^∗^*P* < 0.05 vs. the model group. ^##^*P* < 0.01 and ^#^*P* < 0.05 vs. the CG350 group).

The expression of IL-1*β* and TGF in the wound site was detected by immunohistochemistry. Immunohistochemical examination of the vital wound is summarized in Figures 11 and 12. On the 5^th^, 10^th^, and 15^th^ day, compared with the control group, the content of IL-1*β* in the model group was high (*P* < 0.01). On the 10^th^ day, compared with the model group, the IL-1*β* expression levels of CG350, CG750, and the positive groups decreased significantly (*P* < 0.01). Compared with the CG350 group, the IL-1*β* expression levels of the model, RG, CG150, and CG750 groups increased significantly (*P* < 0.05). On the 15^th^ day, compared with the model group, the IL-1*β* expression levels of CG350 and the positive group decreased significantly (*P* < 0.01). There was no significant difference between CG350 and the positive group (*P* > 0.05). Compared with the CG350 group, the IL-1*β* expression levels of the model, CG150, RG, and CG750 groups increased significantly (*P* < 0.05).

In terms of TGF expression, on the 5^th^, 10^th^ and 15^th^ day, compared with the control group, the content of TGF in the model group was high (*P* < 0.01). On the 5^th^ day, compared with the model group, the expression of TGF in the CG350 group and positive group was significantly higher (*P* < 0.01). Compared with the CG350 group, the TGF content of RG, CG150, and model groups was decreased (*P* < 0.05). On the 10^th^ day, compared with the model group, the TGF content of CG350 and positive groups was increased (*P* < 0.05). Compared with the CG350 group, the TGF content of RG, CG150, CG750, and model groups was decreased (*P* < 0.01). On the 15^th^ day, compared with the model group, the TGF expression of the CG350 group and positive group increased significantly (*P* < 0.01). Compared with the CG350 group, the TGF content of RG, CG150, CG750, and model groups was decreased (*P* < 0.05).

After scald injuries, a natural repair process is initiated, consisting of hemostasis, inflammation, proliferation, and remodelling [35]. High levels of proinflammatory cytokines and mediators, such as IL-1*β*, delayed wound healing [36]. TGF is the primary growth factor responsible for epithelial-mesenchymal transitions and has a generous contribution to skin fibrosis [37].

Our study results corroborated that CG350 had a repair effect on scalded skin. More notably, CG350 significantly downregulated the expression of IL-1*β* and upregulated the expression of TGF to improve scald wound healing.

## 4. Discussion

CG is commonly used to treat scalds and has the effects of secretion absorption, wound healing, and muscle regeneration [38]. According to traditional trait requirements, CG needs to be calcined to be opaque, brittle, and reddish [39]. During calcination, colour and texture are essential appearance indicators to control product quality. Microstructure and pore size are generally considered to be mineralogical properties related to the exudate absorbing capacity of CG. When scalds occur, wounds tend to become infected with microorganisms without treatment [40]. Following wound infection, toxic molecules and metabolites produced by persistent inflammation, bacteria, and immune responses adversely affect wound repair [41]. So far, it is still unclear how the material phase structure of CG impacts the treatment of scald wounds.

In this study, three kinds of phase structures are produced after gypsum calcination: CG150, CG350, and CG750 correspond to bassanite, anhydrite III, and anhydrite II, respectively. CG350 was the most brittle with the largest specific surface area and pore volume, followed by CG150 and CG750. RG's main component is CaSO_4_•2H_2_O; when the calcination temperature rises to 150°C, the internal water molecules spill from the structure, the molecular structure stabilized by RG is destroyed, the spatial structure is unstable, the internal pores increase, and bassanite is generated. A larger crack appeared on the surface of CG150, and the specific surface area and pore volume increased correspondingly. When the calcination temperature was 350°C, anhydrite III without crystallographic water was formed. CG350 showed a larger surface crack, larger specific surface area, and pore volume. Nevertheless, when the temperature increased to 750°C, anhydrite II was formed, the structure was compact, and the specific surface area and pore volume became small. The reason is that high temperature makes CG350 internal sintering, and stable state molecules (or atoms) attract each other [42], thus forming particle binding and powder strength. In turn, the crack, specific surface area, and pore volume of CG750 are smaller than CG350. After scalding, abscesses are easily formed on the skin surface, CG350 has a large pore structure, and its phase structure is anhydrite III, which has a strong water absorption capacity [43]. Thus, CG350 can better absorb exudation in wound abscess, promoting the disappearance of the abscess, which is conducive to scald healing.

Our research group found that the calcium dissolution of RG, CG150, CG350, and CG750 was 3.17%, 3.25%, 4.76%, and 3.19%, respectively. The dissolution of calcium ions in samples is closely related to the pore structure. When CG150, CG350, and CG750 were in contact with water, the water molecules then moved along the channel towards the crystal's interior and interacted with the surrounding ions. After interacting with the surrounding ions, the crystal structure is destroyed, further expanding the contact surface between the crystal and water molecules, accelerating the crystal breaking and forcing Ca^2+^ and SO_4_^2−^ to move to water [44]. CG350 has the most extensive porosity, specific surface area, and pore volume, so calcium ion dissolution is the highest. Studies have shown that calcium ion plays a vital role in scald healing [45] and the anti-inflammatory effect [46]. Cadherin is a kind of cell adhesion glycoprotein with the characteristics of convergence and calcium dependence. It plays an essential role in cell recognition, migration, tissue differentiation, and adult tissue and organ composition [47]. Therefore, it is speculated that the different degrees of dissolution of calcium ions caused by the phase structure may be an important factor causing the difference in biological activity.

Based on the above analysis, the biological activity exerted by CG is closely related to the phase structure, which affects the mineralogical properties and the dissolution of calcium ions. The experimental results showed that CG350, the main phase structure of anhydrite III, exhibited more advantages in texture, pore size, specific surface area, antibacterial, anti-inflammatory, and scald repairing. FTIR and XRD are the most common and reliable methods to determine the phase structure [48, 49]. In the process of calcining gypsum, FTIR and XRD are suggested to identify the phase structure of CG, which is of great significance for the quality control of CG.

## 5. Conclusions

In summary, RG, CG150, CG350, and CG750 are different in mineral properties and biological activities. RG has a dense structure, small pore size, a weak anti-inflammatory effect, but no antibacterial effect, and has almost no effect on the repair of scalds. CG150 has a loose texture, large pore size, and specific surface area and has a unique antibacterial and anti-inflammatory effect, but it has a low repair effect on scalds. CG750 has a compact structure, small pore size and specific surface area, and low antibacterial and anti-inflammatory effects, but it has a specific repair effect on scalds. Only CG350 has good properties in texture, pore size, specific surface area, antibacterial, anti-inflammatory, and scald repair. Our research has proved that, on the one hand, it is reasonable to not use RG for external use to treat trauma. On the other hand, the mineral properties and biological activities of CG are different due to different phase structures; an appropriate temperature should be selected in order to prepare CG with a better curative effect. CG350, namely, anhydrite III, is considered by our research to be the optimal phase structure for CG. Our research provides a reference for determining the best temperature for preparing CG and evaluating CG quality.

## Figures and Tables

**Table 1:** The textural properties of RG, CG150, CG350, and CG750 powder.

Sample	RG	CG150	CG350	CG750
BET surface area (m^2^/g)	1.1215	11.0159	12.5437	3.1734
Pore volume (ml/g)	0.0063	0.0409	0.0438	0.0163
Average pore size (nm)	22.4699	14.8513	13.9672	20.5458

## Data Availability

The data used to support the findings of this study are available from the corresponding author upon request.
